# Methotrexate-Associated Hypersensitivity Pneumonitis After 15 Years of Use: A Case Report and Literature Review

**DOI:** 10.31138/mjr.080225.arc

**Published:** 2025-06-30

**Authors:** Dilara Bulut Gökten, Rıdvan Mercan

**Affiliations:** Division of Rheumatology, Department of Internal Medicine, Tekirdag Namik Kemal University, Tekirdag, Türkiye

**Keywords:** methotrexate, arthritis, rheumatoid, drug hypersensitivity syndrome, hypersensitivity, lung diseases, interstitial

## Abstract

**Objective:**

This report presents a case of methotrexate (MTX)-associated hypersensitivity pneumonitis (HP) after 15 years of use and reviews the literature on its diagnosis, treatment, symptoms, and risk factors.

**Case:**

A 65-year-old female patient with rheumatoid arthritis (RA) presented with shortness of breath and a non-productive cough after 15 years of stable MTX treatment. Chest X-ray revealed bilateral ill-defined infiltrates, and high-resolution computed tomography (HRCT) showed diffuse ground-glass opacities. All diagnostic tests for infection were negative. Suspecting MTX-associated HP, MTX was discontinued, leading to significant clinical improvement.

**Discussion:**

HP is the most common form of pulmonary toxicity associated with MTX. Symptoms typically include dry cough and dyspnoea in 80% of patients. Significant eosinophilia may be observed. Risk factors include age over 60, diabetes, pre-existing lung disease, hypoalbuminemia, RA-related lung involvement, renal dysfunction, male gender, and Daily dose. The diagnosis of MTX-associated HP is a diagnosis of exclusion. Differential diagnosis can be challenging, as it may overlap with other conditions. Although diagnostic criteria have been reported, diagnosis is primarily based on clinical, radiological, and laboratory findings, along with treatment response. Management involves discontinuation of MTX and corticosteroid therapy. While MTX-associated HP generally follows a favourable course with most patients achieving full recovery, reported mortality rates can be as high as 17.6%.

**Conclusion:**

While MTX-associated HP is usually reported within the first years of treatment, it can also occur after prolonged use. Clinicians should consider this possibility in the differential diagnosis, as early detection can result in treatable outcomes.

## INTRODUCTION

Methotrexate, developed in 1948, is an antifolate and antimetabolite drug. It reduces deoxyribonucleic acid (DNA) and ribonucleic acid (RNA) synthesis by causing a deficiency in folate-dependent coenzymes. While it is used at low doses for the treatment of rheumatic diseases, higher doses are employed in the management of malignancies.^1^ Methotrexate (MTX) is widely used in diseases such as psoriasis, polyarticular juvenile idiopathic arthritis, rheumatoid arthritis (RA), and cancer. Low-dose MTX is the cornerstone of RA treatment and has been defined as a first-line therapy by both the American College of Rheumatology (ACR) and European Alliance of Associations for Rheumatology (EULAR). It is also known as an “anchor drug” and is considered one of the safest treatment options.^2^ Moreover, biological DMARDs and tsDMARDs are more effective when combined with MTX.^3^

Although MTX is widely used in clinical practice, one study reported that over 25% of patients receiving this therapy experience coughing, wheezing, breathlessness, or other respiratory symptoms.^4^ Another study further indicated that MTX treatment carries a higher risk of lung disease compared to other DMARDs.^5^ One of the potential lung toxicities associated with MTX, hypersensitivity pneumonitis (HP), was first reported in 1983, and its prevalence among patients receiving MTX has been documented to range from 0.3% to 11.6% depending on differences in study methodologies and diagnostic criteria.^6,7^ MTX-associated HP typically develops within the first years of use; however, cases occurring after long-term treatment have also been reported in the literature[8]. Diagnosis is most commonly made in patients aged 50–60 years, although the youngest reported case in the literature was 38 years old.^9^ MTX-associated HP in patients with RA typically presents with an insidious, non-productive cough, fever, and constitutional symptoms. However, dyspnoea and hypoxemia may develop during the clinical course, potentially requiring hospitalisation.^10^ Although MTX-associated HP typically occurs within the first few years of use, its development after long-term treatment is novel. This report describes a patient diagnosed with MTX-associated HP after 15 years of use and reviews the literature regarding diagnostic criteria, treatment, symptomatology, and risk factors.

## CASE PRESENTATION

**Figure 1.**
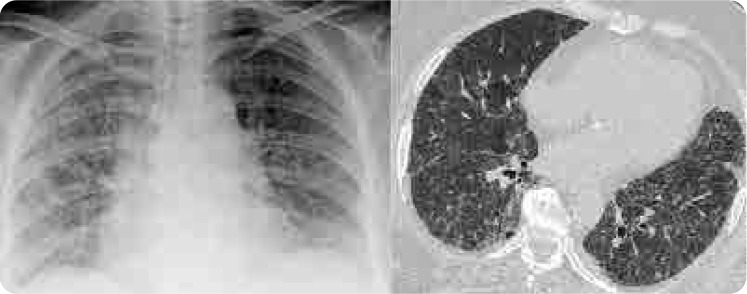
Patient's X-ray and HRCT images at presentation: Chest X-ray showed bilateral infiltrates, HRCT revealed diffuse ground-glass and reticular opacities.

**Figure 2.**
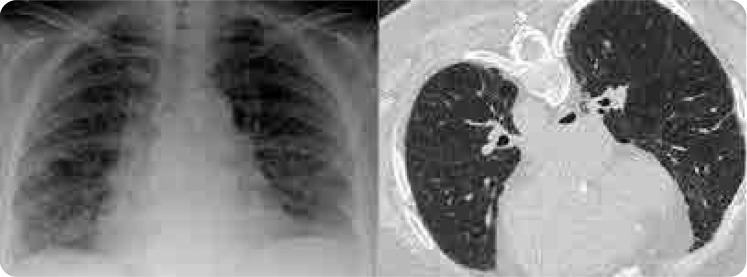
Patient's X-ray and HRCT images after MTX discontinuation: Chest X-ray and HRCT showed significant improvement.

A 65-year-old female patient with no history of smoking presented to the emergency department with progressively worsening shortness of breath and non-productive cough for the past month, along with documented fever for the last 4 days. The patient did not have associated complaints such as chest pain, haemoptysis, palpitations, weight loss, night sweats, or pretibial oedema. The patient had been on stable clinical follow-up for RA for 15 years, receiving 15 mg of weekly MTX and 5 mg of folic acid, along with ramipril and hydrochlorothiazide for hypertension, and 500 mg of metformin for diabetes mellitus (DM) for the past 7 years. She had no history of exposure to dust or toxic gases, and did not report any allergies to medications. The patient had used hydroxychloroquine for about 2 years prior to starting MTX for RA. On physical examination, her temperature was 38.3°C, blood pressure was 120/75 mm Hg, pulse rate was 86 bpm, and respiratory rate was 28 breaths per minute. Upon initial presentation, her oxygen saturation was 83% on room air, but it improved to 94% with a face mask at 10 L/min, FiO2 0.6. Bilateral vesicular breath sounds and fine bibasilar rales were heard, and no pathological findings were noted on other systemic examinations. The patient was then admitted to the ward for close monitoring. Laboratory parameters showed a white blood cell (WBC) count of 9,400/uL (normal level: 4,000–11,000 cells/uL) with a neutrophil percentage of 69.8% (normal level: 40% to 60%). The eosinophil percentage was significantly elevated at 17.4% (0%–6%) The erythrocyte sedimentation rate (ESR) was 110 mm/hour (0–30 mm/h), serum albumin was 3 g/dL (3.4–5.4 g/dl), and lactate dehydrogenase (LDH) level was 255 U/L (125–220 U/L). Other complete blood count values, procalcitonin levels, and BNP (brain natriuretic peptide) were within normal limits. The patient was consulted with a pulmonologist, and differential diagnoses for this condition included community-acquired pneumonia, pneumocystis jirovecii pneumonia (PJP), coronavirus disease (COVID-19), RA-related interstitial lung disease (ILD), allergic alveolitis, and MTX-associated HP. The chest X-ray showed bilateral ill-defined infiltrates, particularly in the middle and lower zones. High-resolution chest CT (HRCT) imaging was consistent with diffuse ground-glass opacities and reticular opacities (**Figure 1**). Influenza and respiratory syncytial virus (RSV) results were negative. Sputum cultures for bacterial pathogens, including acid-fast bacilli (AFB), were negative. The urine Legionella antigen test and the COVID-19 reverse transcription-polymerase chain reaction (RT-PCR) test were also negative. The Beta-D-glucan test was normal, and two sets of blood cultures were negative. The patient was consulted with cardiology, and an echocardiogram performed to assess for potential heart failure was found to be normal. A bronchoscopy was performed on the patient, and bronchoalveolar lavage (BAL) samples were negative for AFB, Gram staining, GenXpert, bacterial and fungal cultures, and PJP. Pulmonary function tests (PFT) showed no obstruction or restriction. Diffusion was mildly reduced, while maximum voluntary ventilation was normal. The patient had no prior diagnosis of RA-ILD, and it was ruled out based on acute clinical presentation and pulmonary function tests. Following the investigations, MTX-associated HP was suspected, and MTX treatment was permanently discontinued. Empirical antibiotic therapy was initiated, along with a daily intravenous hydrocortisone treatment of 60 mg. Clinical improvement was notably observed after 2 weeks of follow-up. After steroid treatment, the eosinophil percentage decreased from 17.4% to 1.2%. The empirical antibiotics were discontinued, intravenous corticosteroids were switched to oral therapy, and the dosage was gradually reduced. After clinical improvement, the patient was discharged on the 17th day of hospitalisation with an oxygen saturation of 96% on room air. Four months later, X-ray and HRCT showed significant improvement (**Figure 2**). The patient has been stable for 6 months in both clinical and laboratory parameters and is being followed with leflunomide 10 mg/day for RA. Written informed consent was obtained from the patient for the publication of this case review and the accompanying images.

### Search strategy and literature review

A web-based search was conducted using the PubMed, Scopus, and DOAJ databases with the keywords methotrexate, hypersensitivity, and hypersensitivity pneumonitis. The search was limited to English-language literature. Additionally, the reference lists of the included articles were examined to identify other relevant publications. Recommendations for writing narrative reviews and case-based review standards were followed.^11,12^ After the literature review, a delineation of similarities and differences was performed, leading to the development of a comparative discussion.

## DISCUSSION

Methotrexate, used as an anti-inflammatory and immunomodulating agent in the course of RA, is discontinued in 1 in 100 patients per year due to associated pulmonary toxicity.^13^ Manifestations of MTX-induced pulmonary toxicity include HP, interstitial pulmonary fibrosis, non-cardiogenic pulmonary oedema, pleuritis and pleural effusion, pulmonary nodules, and cough.^14^ In the literature, some studies have found a weak association between the development of MTX-associated ILD in patients with rheumatic diseases,^15^ while other studies have emphasised the importance of evaluating ILD in individuals receiving MTX within this patient group. Additionally, it has been reported that airway abnormalities such as bronchial wall thickening, air trapping, bronchiectasis detected by HRCT are more common in RA patients.^16,17^ In a study by Pelechas and colleagues, 43 RA patients and 18 healthy individuals were included, and it was reported that ground-glass opacities seen on HRCT were observed exclusively in RA patients. In terms of determining whether MTX truly causes ILD, the authors recommended performing HRCT scans on all patients before starting MTX therapy.^18^

HP is the most common form of pulmonary toxicity associated with MTX.^19^ The symptoms typically include dry cough and dyspnoea in 80% of patients, with fever reported in more than 60% of cases, as seen in this patient. When examining the laboratory parameters, some authors have noted that, in addition to the significant eosinophilia observed in this case, mild peripheral eosinophilia may be present in 25–40% of patients with MTX-associated HP.^7^ Furthermore, some case series have reported a decrease in peripheral clonal lymphocytes, with normalisation of this count after treatment unlike in this case.^20^ While these changes in laboratory parameters and clinical findings are significant in daily practice, given the rarity of MTX-associated HP, they need to be supported by larger studies.^21,22^

The variable incidence of HP, typically occurring within the first year of use, may suggest that the disease is an idiosyncratic immune reaction rather than a dose-dependent toxic injury.^8^ It is a hypersensitivity alveolitis characterised by T cell-mediated (CD4 and CD8) proliferation and the proliferation of type 2 alveolar cells, resulting in cytokine release.^23^ Although risk factors contributing to the development of the disease have been identified, the exact extent to which these factors contribute to the clinical manifestation remains unclear. A review of the literature on the causes of the disease highlights the following risk factors for MTX-associated HP: age over 60, hypoalbuminemia, DM, initiation of second-line RA therapy, RA-related lung involvement, renal dysfunction, male gender, high Health Assessment Questionnaire (HAQ) score, decreased pain Visual Analog Scale (VAS) score, preexisting lung disease, and daily MTX dose rather than weekly dose. There are also some views suggesting that genetic and environmental factors may contribute to the development of the disease.^7, 24^ In the current case, the risk factors included DM, age, hypoalbuminemia, and prior use of DMARDs (**Table 1**).

**Table 1.** Risk factors for MTX-associated HP as reported in the literature.^7,39^

**Risk factors**
Pre-existing lung disease
Age >60
Hypoalbuminemia
Chronic kidney failure
Diabetes mellitus
High HAQ and low VAS scores
Genetic factors (HLA-A31:01)
Environmental factors (latitude)
Prior DMARD use
Male gender

The diagnosis of MTX-associated HP is considered a diagnosis of exclusion. Differential diagnosis can be challenging, as it may overlap with conditions such as PJP, viral and atypical pneumonias, and ILD due to RA (RA-ILD).^18,25^ In the absence of a severe clinical presentation, a significant clinical response to MTX discontinuation and corticosteroid treatment may be sufficient for diagnosis. However, in cases with severe clinical manifestations, clinical and radiological criteria are used. Chest X-ray findings are nonspecific. Especially in the early stages of the disease, chest X-ray findings can be normal. Bilateral acute alveolar infiltrates and interstitial lung markings may be observed, either combined or separately.^26^ On HRCT, ground-glass opacities and centrilobular nodules may be seen.^27^ Similar to the current case, other tests such as BAL or PFT may also be performed for diagnostic purposes. BAL is particularly useful in excluding other infections that could be causing the clinical picture.^7^ Although lung biopsy is 90% sensitive, it can lead to various complications and looking at the literature from this perspective, it has been observed that in most cases, lung biopsy or bronchoscopy is not required.^28^

For diagnosis, the criteria set by Searles and McKendry are commonly referenced in the literature. The major criteria include histopathological evidence of HP without signs of pathogenic organisms, radiological findings of pulmonary interstitial or alveolar infiltrates, and negative blood and initial sputum cultures. The minor criteria include shortness of breath lasting less than eight weeks, non-productive cough, oxygen saturation ≤ 90% on room air upon initial assessment, a diffusing capacity of the lungs for carbon monoxide (DLCO) ≤ 70% of the predicted value for age, and a WBC count ≤ 15,000 cells/mm^3^.^29^ According to these criteria, a definite diagnosis requires one major criterion with three minor criteria (or) two major criteria and three minor criteria, while a possible diagnosis requires two or three major criteria with two minor criteria. In the current case, two major criteria and four minor criteria are present, making a definite diagnosis of MTX-associated HP. Other diagnostic criteria reported in the literature have been outlined by Chikura and colleagues, who evaluate patients from clinical, laboratory, infectious, radiological, and histopathological perspectives. According to their criteria, the clinical domain includes the onset of dyspnoea, whether acute or subacute, and a non-productive cough. In the laboratory domain, an oxygen saturation (SaO₂) of less than 90% on room air and negative blood and sputum cultures are mandatory. Radiologically, the presence of diffuse interstitial abnormalities, bilateral patchy ground-glass opacities, and nodular lesions on HRCT are considered significant. From a histopathological standpoint, findings such as lymphocytic infiltration, diffuse alveolar damage, and BAL findings, including lymphocytosis greater than 30% and/or an increased CD4/CD8 T-cell ratio, are noted. Finally, the treatment domain includes significant symptomatic improvement after the discontinuation of MTX, with or without corticosteroid treatment.^28^ Based on these criteria, 5/8 criteria are classified as definite and 4/8 criteria as likely. According to these diagnostic criteria, the current case is considered as a definite diagnosis of MTX-associated HP as well. However, the validation of these commonly used diagnostic criteria in current practice has not been adequately performed, and it is not recommended to strictly adhere to the criteria in every patient. Even when diagnostic criteria are available, it is important to differentiate MTX-related HP from other potential causes of lung damage in clinical practice, such as RA-ILD, infections, and other drug-induced lung diseases (e.g., hydrochlorothiazide, which may also induce non-cardiogenic lung oedema/lung opacities).^30,31^ There are several distinguishing factors that can help differentiate these entities in clinical practice, and these differences are summarised in **Table 2**.

**Table 2.** Some distinguishing features of conditions that may cause lung damage in RA patients.^7,40–44^

**Feature**	**MTX-HP**	**RA-ILD**	**Infection**	**Drug-induced (e.g., hydrochlorothiazide)**
Onset	Acute/subacute	Insidious	Acute	Acute/subacute
Cough	Non-productive	Non-productive	Productive	Variable
Fever	Common	Rare	Common	Rare
Eosinophilia	Frequent	Absent	Variable	Possible
HRCT Findings	Ground-glass, reticular opacities	Fibrosis, honeycombing, ground-glass, reticular opacities	Consolidation, nodules	Ground-glass, diffuse infiltrates
Response to MTX discontinuation	Significant improvement	No improvement	Variable	Improvement

RA-ILD: rheumatoid arthritis associated interstitial lung disease, MTX-HP: methotrexate-associated hypersensitivity pneumonitis; HRCT: high-resolution chest computed tomography.

A literature review on the treatment of MTX-associated HP reveals that the primary approach includes immediate discontinuation of MTX, along with the use of corticosteroids and cyclophosphamide.^32,33^ Tocilizumab (TCZ), which is effective as monotherapy in RA, has also been reported to be beneficial in the treatment of HP.^34^ In the current case, MTX was immediately discontinued after the hospital admission, and corticosteroids were used in the treatment, resulting in clinical and radiological improvement. This has been crucial in confirming the diagnosis and excluding RA-associated ILD and other bacterial or fungal infectious aetiologies. In this case, the patient’s lung findings improved after the discontinuation of MTX; however, in some cases, full recovery may not be evident on HRCT. As mentioned above, based on the article by Pelechas et al., ground-glass opacities already observed on HRCT in RA patients may hinder full recovery.^18^ Since MTX-related HP is rarer than ILD and airway abnormalities commonly seen in the course of RA, it is important to consider this distinction. While the patient’s pulmonary condition is primarily attributed to MTX in this case, it is crucial to also consider and discuss the alternative possibility that RA-related ILD could be an underlying cause. RA-related ILD is a well-known extra-articular manifestation of RA and can present with similar pulmonary symptoms, such as dyspnoea and ground-glass opacities. While MTX is a common contributor to pulmonary toxicity, its association with ILD may not be the sole cause. Therefore, differentiating between MTX-induced lung injury and RA-related lung involvement is essential for accurate diagnosis and treatment in routine clinical practice.

Regarding prognosis, although the literature reports that MTX-associated HP generally has a favourable course with most patients fully recovering, some cases have shown a mortality rate as high as 17.6%.^35^ In a study by Chikura and colleagues, it was reported that the mortality rate was higher in cases of HP that developed within the first month after the initiation of MTX treatment.^36^ In cases where MTX was re-administered, pulmonary damage recurred, and some cases resulted in death. However, there are also reports in the literature of cases where MTX was reintroduced without any complications.^37,38^

The first limitation of this study is that findings from case-based studies cannot be generalised, as generalisation requires both a confirmed cause-effect relationship and a representative population to which the findings apply. Another limitation is that case studies often focus on rare and atypical conditions, potentially diverting attention from more common diseases and issues. Additionally, the authors reviewed only English-language literature, and the inherent limitations of the review may affect the validity of the main messages. Despite these limitations, this study contributes new insights to the existing literature on MTX-HP by documenting a rare case where the condition developed after 15 years of treatment, contrasting with most previously published cases in which MTXHP emerged within the first few years of use. The report emphasises the importance of a diagnosis of exclusion, supported by a thorough diagnostic process, including laboratory, radiological, and BAL findings. Furthermore, it underscores the necessity of long-term vigilance in patients on MTX therapy, particularly for recognising the condition even after many years of stable treatment, thereby broadening the scope of previously established knowledge.

## CONCLUSION

Although MTX-associated HP is typically reported to occur within the first few years of MTX use, as seen in this case, it can also develop after prolonged use. Clinicians should remain aware of this possibility and include it in the differential diagnosis, as early detection can lead to treatable outcomes. While diagnostic criteria are not definitive, clinical, laboratory, and radiological findings should be evaluated together for an accurate diagnosis. Considering alternative diagnoses and adopting a multidisciplinary approach is essential in managing MTX-related HP, as it ensures a comprehensive evaluation of potential causes, facilitates accurate diagnosis, and enables tailored treatment strategies involving pulmonologists, rheumatologists, and other specialists. Future research should focus on identifying predictive biomarkers and refining diagnostic criteria to enhance early detection and improve patient outcomes.

## AUTHOR CONTRIBUTIONS

All co-authors bear full responsibility for the integrity and accuracy of all aspects of the work.

**DBG:** Contributed to the conception and design of the work, as well as the acquisition, analysis, and interpretation of data. Drafted the manuscript and critically reviewed it for important intellectual content. Contributed to the final approval of the version to be published. Ensured that any questions related to the accuracy or integrity of any part of the work were appropriately investigated and resolved.

**RM:** Contributed to the conception and design of the work and critically reviewed it for important intellectual content. Contributed to the final approval of the version to be published and ensured that any questions related to the accuracy or integrity of any part of the work were appropriately investigated and resolved.

## DISCLAIMERS

Names of commercial editing agencies and agents involved in the writing/editing: None.

Any use of AI for writing and editing: None.

No part of this manuscript, including the text and graphics, are copied or published elsewhere in whole or in part.

## CONFLICT OF INTEREST

The authors declare no conflict of interest.
